# Mechanistic Basis of Super-Infection: Influenza-Associated Invasive Pulmonary Aspergillosis

**DOI:** 10.3390/jof8050428

**Published:** 2022-04-22

**Authors:** Keven Mara Robinson

**Affiliations:** Division of Pulmonary, Allergy, and Critical Care Medicine, Department of Medicine, University of Pittsburgh Medical Center; Pittsburgh, PA 15213, USA; robinsonkm@upmc.edu

**Keywords:** influenza, invasive pulmonary aspergillosis, influenza-associated invasive pulmonary aspergillosis, *Aspergillus fumigatus*

## Abstract

Influenza infection is a risk factor for invasive pulmonary aspergillosis in both immunocompetent and immunocompromised hosts. The purpose of this review is to highlight the epidemiology of influenza-associated invasive pulmonary aspergillosis and the mechanistic studies that have been performed to delineate how influenza increases susceptibility to this invasive fungal infection.

## 1. Introduction

*Aspergillus fumigatus (A. fumigatus)* is a fungus that is ubiquitous in the environment, both indoor and outdoor, and conidia are commonly inhaled into the respiratory tract. *A. fumigatus* can cause a spectrum of clinical syndromes ranging from chronic colonization to invasive disease [1]. Invasive pulmonary aspergillosis (IPA) is a severe, life-threatening disease that occurs when aspergillus conidia are inhaled into the respiratory tract and invade airways or lung tissue. More than 200,000 cases of invasive aspergillosis occur each year [2,3]. Immunosuppressed patients with prolonged neutropenia, inherited or acquired immunodeficiency, hematopoietic stem cell transplantation or lung transplantation are populations most well-known to develop IPA. However, IPA also affects immunocompetent patients; at-risk groups include patients with chronic obstructive pulmonary disease, the critically ill, and those with sepsis [1,4]. Other risk factors include chronic kidney disease, liver failure, diabetes, HIV, autoimmune diseases, burns and malnutrition. A recently confirmed risk factor for IPA in critically ill patients is influenza infection [5,6,7,8,9].

## 2. Epidemiology

Influenza predisposes to secondary bacterial or fungal infections, commonly known as super-infections. IPA has been associated with influenza since the 1950s with case reports characterizing IPA as a complication of influenza. Following the 2009 influenza H1N1 pandemic, multiple epidemiologic studies have confirmed that IPA is a complication of influenza in critically ill patients. A multi-center retrospective cohort study examined infections among adult patients admitted to intensive care units between 2009–2016 in Belgium and the Netherlands [5]. This study identified influenza as an independent risk factor for IPA. In this case, 19% of influenza-infected patients were diagnosed with IPA following admission to the intensive care unit. IPA was associated with 51% mortality (90-day) versus 28% in those without IPA [5]. Other studies have supported these observations of influenza as a risk factor for IPA. A retrospective observational study conducted in Belgium from 2009–2011 identified 23% of patients hospitalized with influenza had IPA [10] and a multi-center retrospective observational study in the Netherlands during the 2015–2016 influenza season found IPA in up to 16% of influenza-infected patients [6]. Data collected from critically ill patients in China from two influenza seasons during 2017–2019 observed an incidence of influenza-associated IPA of 31% and mortality in those patients of 58% [11]. Data collected from critically ill patients in Taiwan from 2015–2016 demonstrated an incidence of influenza-associated IPA of 17% and mortality in those patients of 66% [9]. Another study from Taiwan from 2016–2019 observed a prevalence of influenza-associated IPA of 11.2% [12]. A retrospective cohort study conducted in France between 2009–2018 suggested that 5.3% of patients with influenza may be super-infected with aspergillus [13]. Studies in Canada (2014–2019) and Spain (2009–2015) found an incidence of influenza-associated IPA of 7.2% [14,15]. A retrospective cohort study conducted in the United States examining influenza-infected patients between 2005–2014 found that 0.17% also had IPA. However, this study looked at all hospitalizations related to influenza and not just patients who were critically ill [16]. Notably, variable incidence is seen across studies from different areas of the world ranging from 5–31% excluding the aforementioned study performed in the United States. The heterogeneities between studies (Table 1) may reveal disparities within geographical sites and seasonal variation of influenza viruses, but also methodological differences in the criteria for IPA diagnosis and the clinical management of influenza infections, such as corticosteroids and antivirals, in critically ill patients.

## 3. Diagnosis and Treatment Considerations

Worldwide, annual influenza epidemics result in 3 to 5 million cases of severe influenza and up to 500,000 deaths [17]. Influenza-associated IPA has been described following multiples subtypes of influenza A and influenza B infections [18]. Influenza-associated IPA is difficult to diagnose and although it is estimated that more than 200,000 cases of invasive aspergillosis occur each year, likely representing only 50–65% of actual cases [19]. Although antifungal therapies have improved, treatment is still limited by lack of early diagnosis, fungal identification, route of administration and spectrum of activity [20,21]. Invasive aspergillosis has a 50% mortality rate if diagnosed and treated but is close to 100% lethal if undiagnosed [19]. In 2020, international experts proposed a case definition for influenza-associated IPA that included entry criterion of a patient requiring ICU admission for respiratory distress with a temporally related positive influenza test, either PCR or antigen [22]. Criterion for the diagnosis of proven influenza-associated IPA includes histologic evidence of invasive fungal elements and aspergillus growth on culture or a positive tissue aspergillus PCR. Criterion for the diagnosis of probable influenza-associated IPA includes pulmonary infiltrates and at least one of the following: serum galactomannan (GM) index > 0.5, bronchoalveolar lavage (BAL) GM index > 1, positive BAL culture, or a cavitating infiltrate with either a positive sputum or tracheal aspirate culture. In patients without pulmonary infiltrates but with tracheobronchitis, criterion for the diagnosis of probable influenza-associated IPA includes airway plaques, pseudomembranes or ulcers and at least one of the following: serum galactomannan (GM) index > 0.5, BAL GM index > 1, positive BAL culture, positive sputum or tracheal aspirate culture, or hypal elements consistent with apergillus [22]. The authors suggest that clinicians should not distinguish between proven and probable disease, but these differences should be considered in clinical trials [12,22]. Adopted use of the Amsterdam influenza-associated IPA criteria may reconcile some of the differences observed in prior epidemiologic studies.

Recently published data suggests that influenza-associated IPA should be considered early in the course of influenza infection in critically ill patients in order to perform early diagnostic testing and diagnosis with the goal of early treatment. In Schauwvlieghe and colleagues’ study that identified influenza as an independent risk factor for IPA, the median time from admission to the intensive care unit to the diagnosis of IPA was 3 days [5]. In a recent randomized, open-label trial using posaconazole as antifungal prophylaxis in critically ill influenza-infected patients, 71% of influenza-associated IPA was diagnosed within 48 h of admission [23]. Wauters et al., identified the median time from admission to the intensive care unit to the diagnosis of influenza-associated IPA as 3 days and the median time from influenza diagnosis to influenza-associated IPA diagnosis as 2 days [10]. Van de Veerdonk et al., showed that patients who survived influenza-associated IPA received antifungal therapy earlier than non-survivors (median time 2 days versus 9 days post influenza diagnosis) [6]. These data suggest that influenza-associated IPA typically presents early in the course of influenza infection among patients admitted to the intensive care unit and that rapid antifungal treatment decreases mortality.

In addition to the rapid diagnosis and treatment of influenza-associated IPA, other therapies should be carefully considered when caring for critically ill influenza-infected patients. Corticosteroid use has been shown to be a significant risk factor for the development of influenza-associated IPA [10,15,24] and is associated with a poor outcome during influenza infection [25,26]. A heightened level of awareness for the possibility of influenza-associated IPA is likely warranted in patients with influenza that receive corticosteroids. Posaconazole prophylaxis has been studied in a randomized, open-label, proof-of-concept trial in critically ill patients with influenza for prevention of influenza-associated IPA. However, the primary endpoint was the incidence of influenza-associated IPA in patients who were not diagnosed with influenza-associated IPA within 48 h of admission to the intensive care until and 71% of influenza-associated IPA was diagnosed within the first 48 h, excluding them from the modified intention-to-treat population. The authors were unable to draw any definite conclusions regarding posaconazole use as prophylaxis against influenza-associated IPA [23] and antifungal prophylaxis against influenza-associated IPA warrants additional investigation.

## 4. Mechanistic Studies

As the body of knowledge of the epidemiology of influenza-associated IPA has grown and continues to be investigated, additional mechanistic studies have been published. The first line of host defense against airborne aspergillus conidia requires a healthy respiratory epithelium and effective mucociliary clearance, both of which can be damaged by influenza [27]. Interestingly, aspergillus tracheobronchitis has been described in patients with severe influenza and IPA potentially related to damage of the respiratory epithelium by influenza, allowing for invasion by aspergillus [6,28,29].

Local immune dysregulation induced by influenza allows for increased susceptibility to IPA. The innate immune system responds to penetration of the epithelium by aspergillus with macrophage phagocytosis of conidia and recruitment of neutrophils to the airspace. Neutrophils are able to destroy aspergillus hyphae [30]. Using a murine model, our group has shown that post-influenza IPA results in increased fungal burden, viral burden, inflammation, and mortality compared to mice singularly infected with either influenza or aspergillus alone [31]. Alveolar macrophages are decreased in post-influenza IPA compared to mice singularly infected with aspergillus [31]. Both alveolar macrophage depletion and altered function have been implicated to increase susceptibility to post-influenza bacterial pneumonia and may play a role in post-influenza IPA [32,33,34,35,36]. Additionally, although super-infected mice are not neutropenic, neutrophil recruitment to the lung is inhibited as a result of influenza A-induced STAT1 signaling. Neutrophil chemokine ligands CXCL1 and CXCL2 are decreased in super-infected mice. Deletion of STAT1 during post-influenza IPA results in increased airway and lung neutrophilia and increased levels of CXCL1. Eosinophils also contribute to clearance of *A. fumigatus* from the lung [37] and eosinophil levels are also decreased in super-infected mice compared to mice singularly infected with aspergillus, and warrant additional investigation [31].

Recent studies have addressed the effects of viral therapies on post-influenza IPA. Varying results have been observed with neuraminidase inhibitors, whereas endonuclease inhibitors could decrease susceptibility to IPA. Seldeslachts and colleagues have demonstrated, in a murine model of influenza-associated IAPA (IAPA), that early oseltamivir inhibited the development of IAPA and associated morbidity including weight loss, lung pathology including multifocal hyperdense lung lesions and nonaerated lung volume, and fungal burden. Macrophages with intracellular aspergillus conidia were observed in oseltamivir-treated mice on Day 3 post-fungal challenge while this was not observed in non-treated mice. The authors suggested that influenza may lead to immune dysregulation and decreased macrophage phagocytic activity [38]. Dewi and colleagues showed that oseltamivir increased susceptibility of mice to IPA. Oseltamivir inhibited both mouse splenocyte and human peripheral blood mononuclear cell (PBMC) killing capacity of *A. fumigatus* and restoring neuraminidase to human PBMC enhanced the killing capacity of PBMC against *A. fumigatus.* The sialic acid-binding receptor SIGLEC15 was increased in *A. fumigatus*-stimulated PBMCs and silencing of SIGLEC15 inhibited PBMC killing of *A. fumigatus.* When aspergillus conidia were treated with neuraminidase, the same effects of silencing SIGLEC15 were not observed. This study suggests that neuraminidase has a role in antifungal host defense and the authors hypothesize that neuraminidase inhibitors commonly used during influenza infection may predispose to the development of secondary IPA [39]. Additional investigation is needed to differentiate the effects of oseltamivir during singular influenza infection and IPA, and what occurs mechanistically during influenza-associated IPA. In a murine model of influenza-associated IPA, baloxavir marboxil, a cap-dependent endonuclease inhibitor, was shown to improve weight loss, survival, viral clearance, and decreased fungal invasion. Baloxavir marboxil-treated mice had higher levels of alveolar macrophages in bronchoalveolar lavage fluid compared to non-treated mice, which could play a role in fungal burden and fungal invasion during IAPA [40].

## 5. Conclusions

**Figure 1:**
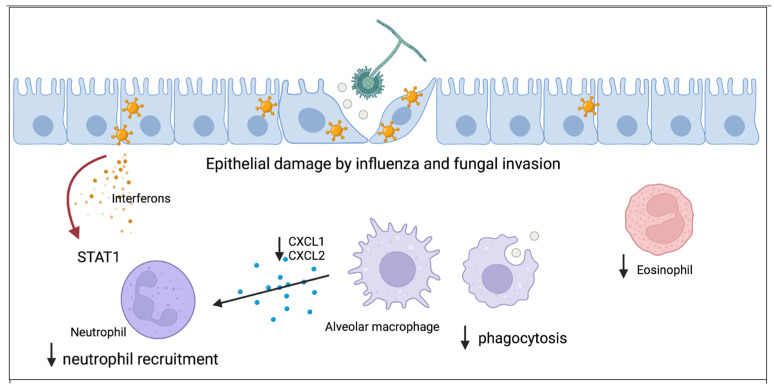
Proposed model for how influenza alters antifungal immunity and increases susceptibility to aspergillus infection. The first line of host defense is healthy respiratory epithelium which can be damaged by influenza and allow for fungal invasion. Resident alveolar macrophages phagocytose aspergillus and both total numbers and function of macrophages is inhibited by influenza. Macrophages secrete cytokines to recruit neutrophils to the lung and influenza inhibits neutrophil recruitment through STAT1 signaling. Eosinophils have antifungal properties and influenza decreases eosinophil levels during invasive pulmonary aspergillosis.

Improving our knowledge of the host immune response to IPA is critical to the advancement of therapies and vaccines. Notably, IPA can also complicate other viral infections such as SARS-CoV-2, respiratory syncytial virus, parainfluenza, and adenovirus [41,42]. Understanding how preceding viral infections, such as influenza, increase susceptibility to IPA will allow for new insights into fungal host defense. Current research has started to elucidate the breaches in host defense that occur during influenza infection allowing for the development of post-influenza fungal super-infection (Figure 1). Existing studies indicate that regulation of innate immune cells such as macrophages and neutrophils will be key to understanding influenza-associated IPA pathogenesis.

## Figures and Tables

**Table 1:** Summary of studies evaluating post—influenza IPA.

References		Location	Influenza Season
Schauwvlieghe, et al. (2018) [5]	Belgium and The Netherlands	2009–2016	**Findings**
Wauters, et al. (2012) [10]	Belgium	2009–2011	19% of influenza-infected patients were diagnosed with IPA, IPA was associated with 51% mortality
van de Veerdonk, et al. (2017) [6]	The Netherlands	2015–2016	23% of influenza-infected patients were diagnosed with IPA
Huang, et al. (2020) [11]	China	2017–2019	16% of influenza-infected patients were diagnosed with IPA
Ku, et al. (2017) [9]	Taiwan	2015–2016	31% of influenza-infected patients were diagnosed with IPA, IPA was associated with 58% mortality
Coste, et al. (2021) [13]	France	2009–2018	17% of influenza-infected patients were diagnosed with IPA, IPA was associated with 66% mortality
Schwartz, et al. (2020) [14]	Canada	2014–2019	5.3% of influenza-infected patients were diagnosed with IPA
Martin-Loeches, et al. (2017) [15]	Spain	2009–2015	7.2% of influenza-infected patients were diagnosed with IPA
Sharma, et al. (2020) [16]	United States	2005–2014	7.2% of influenza-infected patients were diagnosed with IPA
Wu, et al. (2017) [12]	Taiwan	2016–2019	0.17% of influenza-infected patients were diagnosed with IPA

## Data Availability

No new data were created or analyzed in this study. Data sharing is not applicable to this article.
